# The magnitude of adherence to option B plus program and associated factors among women in eastern African countries: a systematic review and meta-analysis

**DOI:** 10.1186/s12889-020-09903-y

**Published:** 2020-11-27

**Authors:** Reta Tsegaye, Werku Etafa, Bizuneh Wakuma, Getu Mosisa, Diriba Mulisa, Tadesse Tolossa

**Affiliations:** 1 School of Nursing and Midwifery, Institute of Health Sciences, Wollega University, Nekemte, Ethiopia; 2 Department of Public Health, Institute of Health Sciences, Wollega University, Nekemte, Ethiopia

**Keywords:** Option B plus, Adherence, Eastern African countries

## Abstract

**Background:**

Despite coverage and benefits associated with the prevention of mothers to child transmission (PMTCT) services, mothers’ adherence to option B plus is still a challenge. Though few primary studies are available on the magnitude of adherence to option B plus and factors associated in Eastern African countries, they do not provide strong evidence in helping policymakers to address suboptimal adherence to option B plus. Therefore, this systematic review and meta-analysis was intended to estimate the pooled magnitude of adherence to option B plus program and associated factors among women in Eastern African countries.

**Methods:**

PubMed, Medline, HINARI, Cochrane library, the Web of Science, and Google Scholar were searched for studies reported on the magnitude of adherence to option B plus among women in Eastern African countries. The search terms used were “option B plus”, “magnitude”, “prevalence”, “PMTCT”, “ART adherence”, “associated factors”, “all lists of Eastern African countries” and their combination by Boolean operators. The effect sizes of the meta-analysis were the magnitude of adherence to option B plus and the odds ratio of the associated factors. STATA/SE V14 was used for statistical analysis, and publication bias was assessed using funnel plots and Egger’s test.

**Results:**

Fourteen studies having total participants of 4883 were included in the systematic review and meta-analysis. Using the random effect model, the pooled prevalence of adherence to option B plus was 71.88% (95% CI: 58.54–85.23%). The factors associated with good adherence to option B plus PMTCT program were partner support (Adjusted odds ratio (AOR) = 4.13; 95% CI: 2.78–6.15), received counseling services (AOR = 4.12, 95% CI: 2.81–6.02), disclosure of HIV status to partner (AOR = 4.38; 95% CI: 1.79–10.70), and clinical stage of HIV/AIDS I/II (AOR = 2.62; 95% CI: 1.53–4.46).

**Conclusion:**

The level of adherence to option B plus program in Eastern African countries was generally sub-optimal. Thus, a coordinated effort is needed to raise the number of mothers to be tested, and early treatment initiation for HIV positive mothers before the disease advances. Furthermore, counseling services for couples on the importance of early treatment initiation and adherence to medications must be given due attention.

## Background

The transmission of Human Immunodeficiency Virus (HIV) from mother to child occurs in three possible periods; this includes prenatal, delivery, and the postnatal period (breastfeeding). Mother-to-child transmission (MTCT) accounts for greater than 10% of HIV infections worldwide and also contributes to more than 90% of HIV infection among infants and young children [1, 2]. Worldwide, there were an estimated 1,400,000 HIV infected women who give birth to about 330, 000 HIV infected babies annually. Among these, sub-Saharan Africa accounts for 91% [3]. In 2018, 1.1 million children aged 0–14 years were living with HIV in East and Southern Africa, in which the main route for HIV transmission was through birth [4].

The World Health Organization (WHO) has introduced different approaches to scale-up prevention of MTCT of HIV. These approaches are Option A, Option B, and Option B plus. In option A, HIV positive pregnant mothers should start triple ARV medication as soon as diagnosed or as early as 14 weeks of gestation and continue until 7 days of postpartum or for life, based on their CD4 count. In option B, HIV positive pregnant mothers should start triple ARV medication as soon as diagnosed or at the early fourteenth week of gestation and continue until childbirth if not breastfeeding or until 1 week after cessation of breastfeeding or for life, based on their CD4 count [5]. The third approach was option B plus which was first conceived and implemented in Malawi in 2011 and is now being used in many countries, including Eastern African countries like Ethiopia, Kenya, Uganda, and Tanzania, to mention a few. This option recommends all HIV positive pregnant women to be given a life-long ART, irrespective of CD4 count starting as soon as diagnosed. Unlike the previous two options, option B plus played a crucial role in early ART initiation and reduction of sexual transmissions among serodiscordant partners [6–8].

A current lifelong treatment by a triple combination of antiretroviral drugs (Option B plus) has registered remarkable success in the PMTCT rate when compared with giving a single dose of zidovudine during pregnancy and labor [9]. Access to counseling and testing of HIV; initiation of lifelong antiretroviral therapy (ART) with support for good adherence; and viral suppression for women living with HIV; safe delivery practices; and access to postnatal antiretroviral (ARV) prophylaxis for infants all contribute to the PMTCT; thus reducing maternal and child mortality [10].

However, despite the wide scale-up, coverage, and benefits associated with PMTCT services, adherence of mothers to the treatment is still a challenge. Recent data show that adequate adherence drops from 75.7% during pregnancy to 53% postpartum among women who meet present ART criteria [11]. Non-compliance to ART medication increases the risk of drug resistance, virologic failure, and progresses the disease to advanced stage, which may lead to an increased risk of mother to child transmission of HIV. Poor adherence is the key reason for low treatment outcomes among women receiving ART. This may speed up the move from first-line regimens to more expensive second-line regimens at the early stage of the disease besides directly affecting women’s well-being [12].

Studies conducted in East African countries revealed the different levels of adherence to option B plus and factors affecting it. These factors include residence, time to reach a health facility, educational status of the mother, access to counseling, partner support, and stage of disease [7, 12–18]. But there is no pooled level of adherence to option B plus to prevent MTCT of HIV in these countries. Thus, this systematic review and meta-analysis was intended to estimate the pooled level of adherence to option B plus program and to estimate the pooled effect of its associated factors in the East African region.

## Methods

### Search strategy

This review was conducted to measure the pooled magnitude of adherence to Option B plus antiretroviral therapy and associated factors among mothers on the PMTCT program in Eastern African countries. A Preferred Reporting Items for Systematic Reviews and Meta-Analysis (PRISMA) checklist was used to conduct the review [19]. To avoid the occurrence of duplication, this study’s protocol was registered in the PROSPERO with the registration number of CRD42019130288 on 02 May 2019. Published and unpublished studies since 2013 were searched from common electronic databases like PubMed, the Web of Science, Medline, HINARI, Cochrane library, and Google scholar. Searches were conducted using terms such as “option B plus”, “magnitude”, “prevalence”, “PMTCT”, “ART adherence”, “associated factors” and “determinants” and all lists of East African countries“ by using Boolean operators like “AND” and “OR”. For instance, the search string we searched from PubMed database was ((((((Option B plus [tw]) OR (PMTCT [tw] OR prevention of mother to child transmission [tw]))) AND (((adherence [tw] OR compliance [tw])) OR (“Advance Directive Adherence”[Mesh] OR “Guideline Adherence”[Mesh] OR “Treatment Adherence and Compliance”[Mesh] OR “Medication Adherence”[Mesh] OR “Patient Compliance”[Mesh]))) AND (((magnitude [tw] OR prevalence [tw] OR distribution [tw])) OR (“Prevalence”[Mesh] OR “Epidemiology”[Mesh] OR “epidemiology” [Subheading]))) AND ((women) OR (“Women”[Mesh] OR “Pregnant Women”[Mesh] OR “Postpartum Period”[Mesh]))) AND Ethiopia Filters: Full text, in the last 10 years, English, Humans.

#### Inclusion and exclusion criteria

This review included articles conducted only in Eastern African countries on the level of adherence to option B plus program among women who were following the option B plus PMTCT program. Articles and grey literature published in English till November 2019, were considered in this meta-analysis. The review has included studies conducted by cross-sectional and cohort study designs. Duplicated reports; article with inconsistent findings; articles from which an Odds Ratio (OR) could not be calculated; articles published in a language other than English; were excluded from the analysis. This systematic review and meta-analysis used the CoCoPop (condition, context, population) framework for question development. The condition was the magnitude of option B plus adherence and associated factors, the context was East African countries and the population (P) was women on option B plus PMTCT program.

### Study outcome

The main aim of this systematic review and meta-analysis was the magnitude of adherence to option B plus PMTCT program. A questionnaire adapted from experience in South Africa was used to measure the level of adherence to option B plus which was primarily designed to measure compliance in resource-limited settings [20]. Primary articles included in the current meta-analysis also used the same scale of measurement. Good adherence to option B plus program was considered if a study participant responded “no” to all four of the questions. However, if the mother responded “yes” to at least one question, she was considered to have poor adherence. The second outcome of the review was the determinants of adherence to the Option B plus program. It was determined using the odds ratio (OR) and calculated based on binary outcomes from the included primary studies.

### Quality assessment and data abstraction

**Fig. 1:**
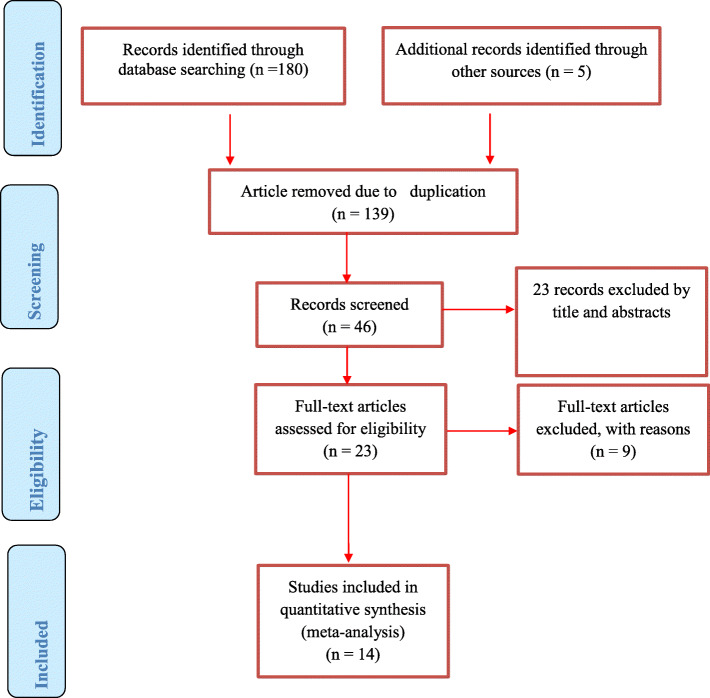
PRISMA flow diagram of included studies in the systematic review and meta-analysis of the adherence to option B plus PMCT program, 2019

We did a critical appraisal by using The Joanna Briggs Institute Meta-Analysis of Statistics Assessment and Review Instrument (JBI-MAStARI) [21]. Two reviewers (DM and RT) reviewed the articles independently and then compared those articles to make sure all available articles are downloaded and selected for inclusion. They also avoided duplication by using endnote, excluded studies by their titles and abstracts, and assured that all articles available and data have been entered into a checklist prepared in Microsoft Excel. Then other reviewer TT and BW arbitrated any inconsistencies between the two reviewers (DM and RT). The systematic review and meta-analysis followed the Preferred Reporting Items for Systematic Review and Meta-Analyses (PRISMA) flow chart to identify and select relevant studies for this analysis (Fig. 1). Reference management software (EndNote X9 Bld 12,062) was used initially to combine articles searched from databases and to remove duplicate search results. Then, we screened and excluded studies by their titles and abstracts. Articles that exist in full-text were identified for the remaining articles. By using predetermined inclusion and exclusion criteria, the eligibility of the studies was evaluated. For the first outcome (magnitude of adherence to option B plus), the data extraction checklist included author name, country (Eastern part of African countries where the study was conducted), year of publication, sample size, study design, and the number of participants with the good adherence to option B plus. For the second outcome (factors associated with adherence to option B plus), the checklist for data extraction contains the name of authors, publication year, countries (the Eastern part of African countries where the study was conducted), study design, sample size, and column of a variable under categories of good and poor adherence to option B plus. We extracted the data in a format of two by two tables and then calculated the log OR based on the results of the initial studies. Disagreements between two independent reviewers were fixed by involving another independent reviewer (BW) after discussion for possible consensus. When articles had inadequate data, corresponding authors of the research articles were contacted through their email address.

### Statistical analysis

After data were extracted from each eligible article, it was imported into STATA/SE windows Version 14 software for analysis. The 95% confidence intervals (95% CI) was used to calculate the effect of the independent variable on option B plus ART adherence status and pooled magnitude of adherence to option B plus using the Der Simonian and Laird random-effects meta-analysis (random-effects model) [22]. In this study to provide a visual image of the data, the pooled effect size and effect of each study with their confidence interval (CI) were estimated by a forest plot.

### Heterogeneity and publication bias

The *p*-values of Cochran’s Q-test and I^2^-test statistics were used to assess heterogeneity across studies. Cochran’s *Q* statistical heterogeneity test was considered statistically significant at *p* ≤ 0.05. The degree of heterogeneity was evaluated by the index of heterogeneity I^2^ statistics. When the values of I^2^ test statistics were 25, 50, and 75%, heterogeneity was assumed to represent low, medium, and high respectively. Due to heterogeneity among studies, we performed a subgroup analysis based on study design. A small study effect was seen by sensitivity analysis and publication bias was evaluated by funnel plot test. Moreover, the statistical presence of publication bias was assessed using Egger’s test.

## Results

In our initial literature search, a total of 180 published and five unpublished articles related to our review title were accessed. From those first searched, 139 were removed due to duplication of records, and the remaining 46 articles were screened for eligibility. From 46 articles, 23 of them were excluded by their title and abstract. Then 23 studies were assessed and screened for eligibility criteria based on the outcome variables related to ART adherence of option B plus among which nine articles were excluded because of non-relevance to the current review. Finally, 14 articles fulfilled all the eligibility criteria and were included in this analysis.

### Overview of included studies

All the 14 articles included in this study were published between 2015 and 2019. A total of 4883 study participants were included in this systematic review and meta-analysis. The smallest sample size was 41 from a study conducted in Ethiopia and the largest sample size was 1150 from a study conducted in Zimbabwe. Three studies were cohort studies while the remaining all were cross-sectional studies. Published studies conducted in Eastern African countries (Tanzania, Uganda, Ethiopia, Zambia, Zimbabwe, and Malawi) have been included.

### The magnitude of good adherence to option B plus PMTCT program

This systematic review and meta-analysis have identified that the magnitude of adherence to option B plus PMTCT program in Eastern African countries ranges from 31.9% (from a study conducted in Malawi) to 95.97% (study conducted in Uganda) (Table 1). Prevalence in this table is to mean that the magnitude of pregnant and laboring women who had good adherence to option B plus PMTCT program based on four measurement questions adapted from the experience in South Africa, which were designed to measure adherence in resource-constrained settings [15]. The primary studies included in this meta-analysis used the same scale of measurement to say good and poor adherence.

**Table 1:** Summary of included studies on the magnitude of Option B plus antiretroviral therapy adherence and associated factors, 2019

Author	Year of publication	Country	Study design	sample size	Prevalence (95%CI)
Zacharius et al. [15]	2019	Tanzania	Cross-sectional	305	45.90 (40.31,51.49)
Tsegaye et al. [12]	2016	Ethiopia	Cross-sectional	191	87.43 (82.73,92.14)
Tesfaye et al. [16]	2019	Ethiopia	Cross-sectional	290	81.38 (76.90,85.86)
Shibabaw et al. [23]	2018	Ethiopia	Cross-sectional	41	95.12 (88.53,101.72)
Mihratu et al. [24]	2018	Ethiopia	Cross-sectional	304	79.61 (75.08,84.13)
Lodebo et al. [25]	2017	Ethiopia	Cross-sectional	215	83.72 (78,79,88.66)
Ebuy et al. [13]	2015	Ethiopia	Cross-sectional	277	87.00 (83,04,90.96)
Schnack et al. [26]	2016	Uganda	Cross-sectional	124	95.97 (92.51,99.43)
Okawa et al. [27]	2015	Zambia	Cohort study	321	82.55 (78.40,86.71)
Haas et al. [28]	2016	Malawi	Cohort Study	765	30.07 (26.82,33.31)
Mwalumuli EO [29]	2017	Tanzania	Cross-sectional	338	58.88 (53.63,64,12)
Erlwanger et al. [30]	2017	Zimbabwe	Cohort study	1150	31.91 (29.22,34.61)
Yazine D et al. [31]	2017	Ethiopia	Cross-sectional	269	75.09 (69.92,80.26)
Mihretu T et al. [7]	2019	Ethiopia	Cross-sectional	293	82.59 (78.25,86.94)
Over all with weights from random effect					71.88 (58.54,85.23)

**Fig. 2:**
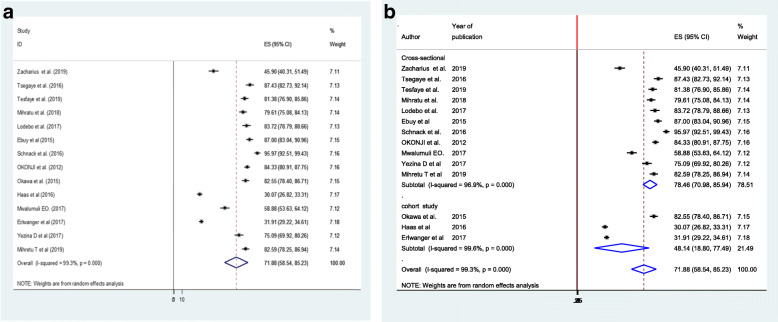
**a** Forest plot of the pooled magnitude of adherence to option B plus PMTCT program, 2019. **b** Sub-group analysis of studies included in the meta-analysis on the magnitude of adherence to option B plus PMTCT program, 2019

As shown in (Fig. 2a), the overall point estimate of the magnitude of good adherence to option B plus PMTCT program in Ethiopia was 71.88% (58.54,85.23%). The subgroup analysis showed that the magnitude of good adherence to option B plus PMTCT program was 78.46% (95%CI: 70.98–85.94) for the cross-sectional studies and 48.14% (95%CI: 18.80–77.49%) for the cohort studies (Fig. 2b). However, there is evidence of heterogeneity between cohort studies. This could be due to the difference in the enrollment of study participants among these studies and the difference in the estimation of prevalence. In the study conducted by Okawa et al 2015, the median time of enrollment of women for the study was 10 weeks and a prevalence of adherence was estimated during pregnancy, 1 week postpartum, 6 weeks postpartum, and 24 weeks postpartum. In the study conducted by Haas et al *2016,* pregnant women were enrolled (unspecified week of gestation) and a prevalence estimate was conducted during pregnancy (unspecified week of gestation) and breastfeeding. In the study conducted by Erlwanger et al *2017*, pregnant women were enrolled during the first trimester, and prevalence was estimated at the same time.

### Publication bias

**Fig. 3:**
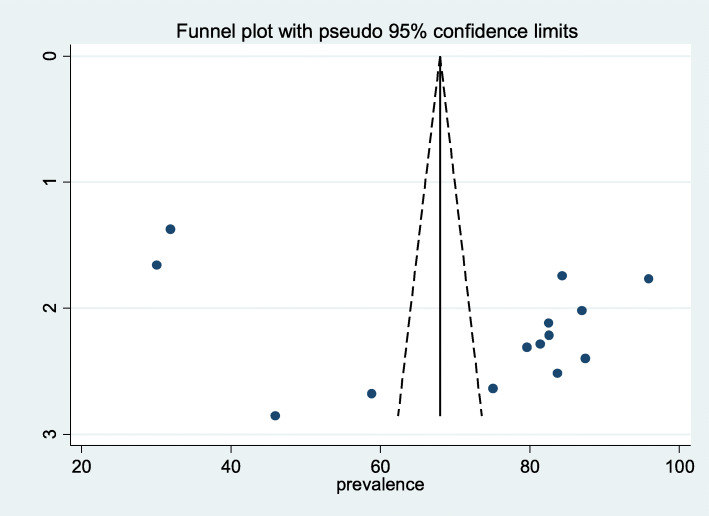
Funnel plot with 95% confidence limit of the pooled magnitude of adherence to option B plus, 2019

To assess the presence of publication bias, funnel plot, and Egger test at 5% significant level were computed. Visual interpretation of the funnel figure looks asymmetrical and may show the presence of publication bias. However, statistical evidence of Egger’s test revealed that there is no publication bias (*P* = 0.103) (Fig. 3).

To identify single study influence on the overall meta-analysis, a sensitivity analysis was performed using a random-effects model and the result showed there was no strong evidence for the effect of single study influence on the overall meta-analysis.

## Factors related to adherence to option B plus program

### Association between adherence to option B plus PMTCT program and partner support

**Fig. 4:**
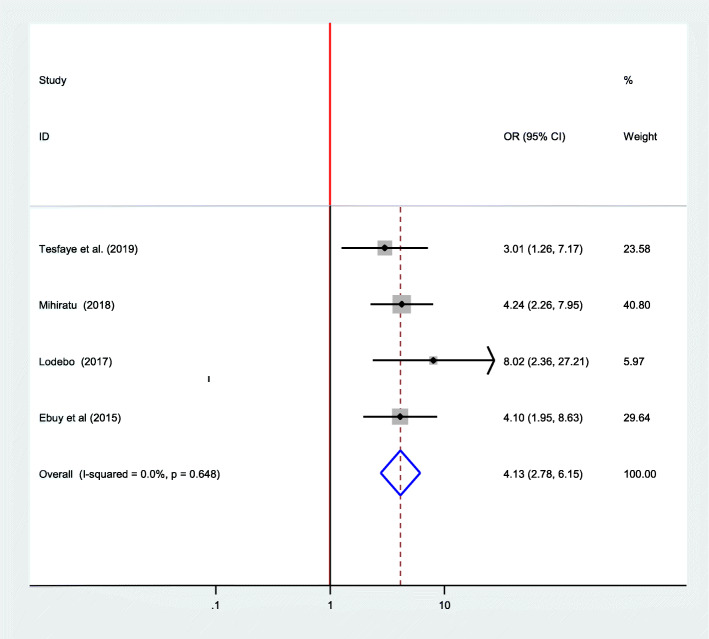
Forest plot of the association between partner support and adherence to option B plus PMTCT program, 2019

Regarding the presence of partner support, four articles conducted in Ethiopia were included [7, 16, 24, 25]. According to those research findings, presence of partner support is positively associated with adherence to option B plus PMTCT program [7, 13, 24, 25]. The pooled effect from this meta-analysis also showed that partner support is positively associated with adherence to option B plus. This means those women who have partner support are four times more likely to adhere to option B plus PMTCT program compared to those who have no partner support (OR = 4.13; 95% CI: 2.78–6.15). The fixed-effect model was used because, the included studies did not exhibit heterogeneity (I^2^ = 0.00%, *p* = 0.648) (Fig. 4).

### Association between adherence to option B plus PMTCT program and disclosure status

**Fig. 5:**
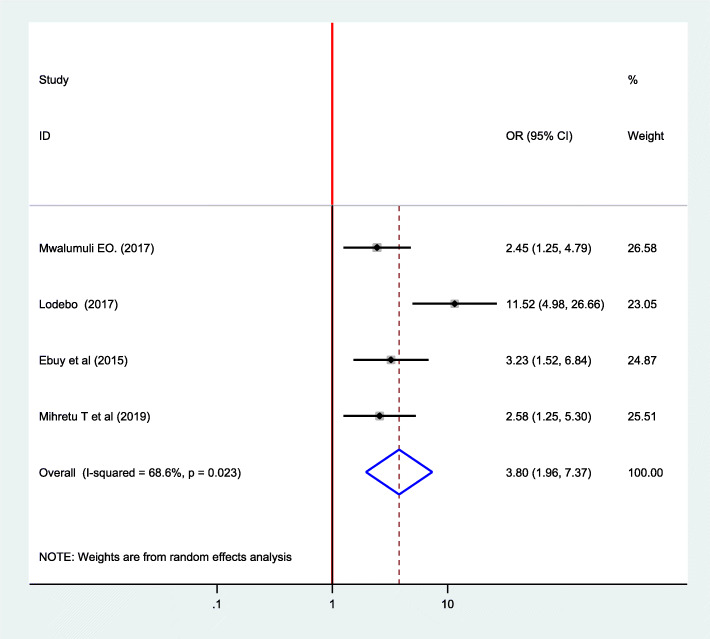
Forest plot of the association between disclosure of HIV serostatus and adherence to option B plus PMTCT program, 2019

Regarding disclosure status, one study conducted in Tanzania [29] and three in Ethiopia [7, 13, 25] were included. These studies identified that disclosing their HIV serostatus to their partner or family increases the level of adherence to option B plus PMTCT program. The Meta-regression by random effect model indicated that those mothers who disclosed their HIV serostatus were 4.38 times more likely to adhere to option B plus PMTCT program compared to their counterparts. (OR = 4.38; 95% CI: 1.79 10.70) (I^2^ 76.4%, *p* = 0.014) (Fig. 5).

### Association between adherence to option B plus PMTCT program and received counseling

**Fig. 6:**
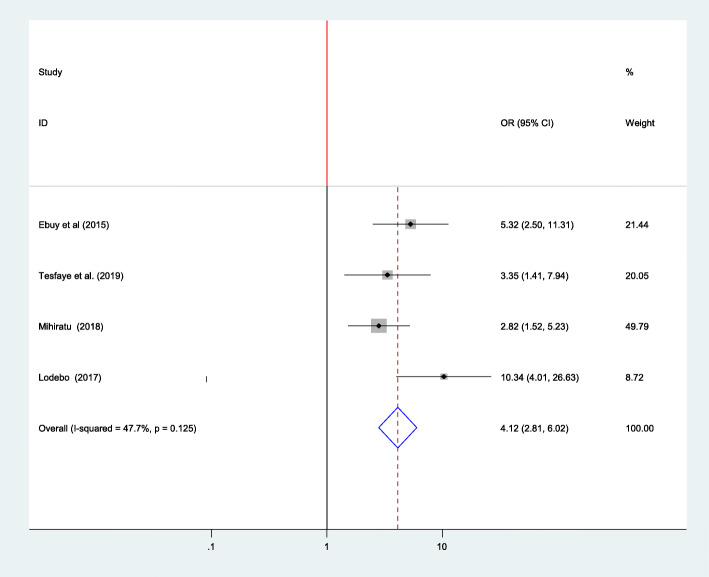
Association between adherence to option B plus PMTCT program and received counseling, 2019

Four studies were included in the analysis to estimate the pooled effect of counseling on adherence to option B plus among women. Using the fixed-effect model, the pooled estimate has found out that women who received counseling about ART medications and adherence were 4.12 times more likely to adhere to option B plus PMTCT program compared to non-counseled women (OR = 4.12, 95% CI:2.81–6.02) [7, 13, 16, 25]. The heterogeneity among the four studies used to estimate the pooled estimate among women was (I^2^ = 47.7%, *p* = 0.125) (Fig. 6).

### Association between adherence to options B plus PMTCT program and stage of HIV/AIDS

**Fig. 7:**
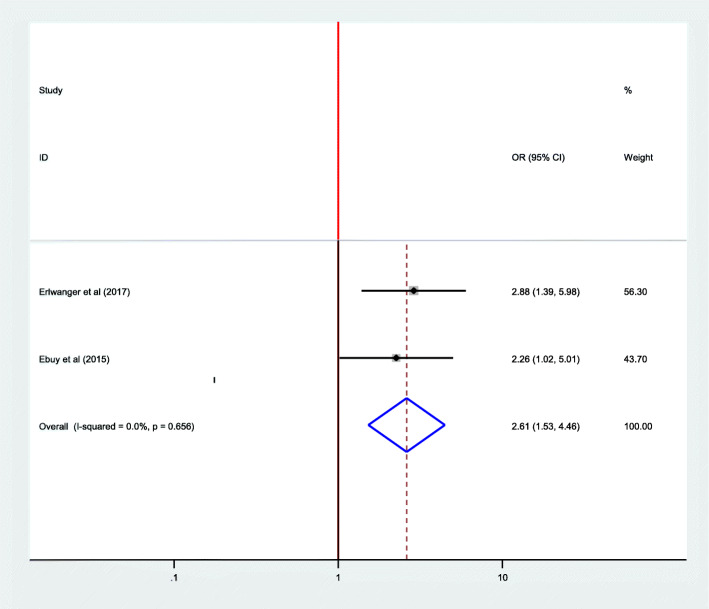
Association between adherence to options B plus PMTCT program and stage of HIV/AIDS, 2019

Two studies were included in the analysis to estimate the pooled effect of stage of HIV/AIDS on adherence to option B plus among women. From these, one study conducted in Zimbabwe [30] showed significant association while a study conducted in Ethiopia [13] didn’t show significant association. The overall pooled estimate of a stage of HIV/AIDS also revealed that there is a significant difference between HIV/AIDS stage I/II and stage III/IV on adherence to option B plus (OR = 2.62; 95% CI: 1.53–4.46). The fixed-effect model was used for this regression because it did not exhibit heterogeneity (I^2^ = 0.00%, *P* = 0.656) (Fig. 7).

### Association between adherence to option B plus PMTCT program and mother’s educational status

**Fig. 8:**
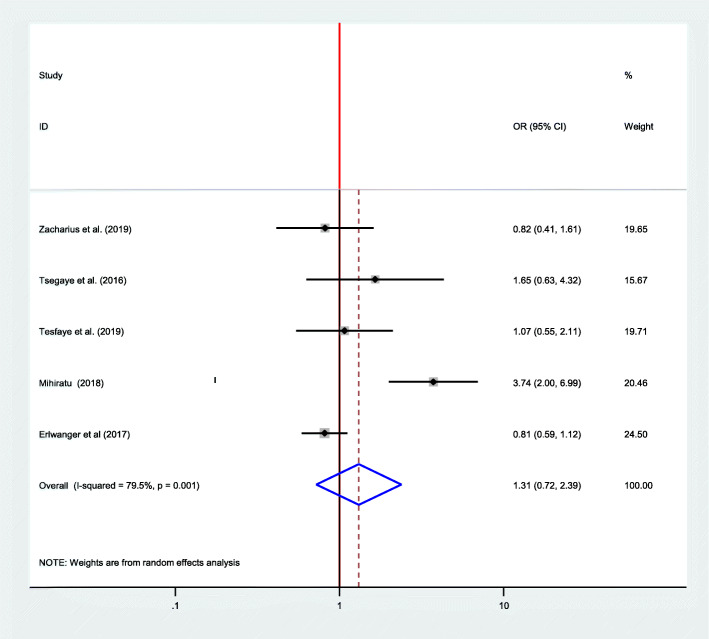
Forest plot of the association between educational status and adherence to option B plus PMTCT program, 2019

Five articles have been included in meta-regression to see the relationship between the educational status of the mothers and adherence to option B plus PMTCT program [12, 13, 16, 30]. From these studies, only one study has shown a significant association [16]. However, the pooled effect showed that there was no significant association between the educational status of the women and adherence to option B plus (OR = 1.31; CI 0.72–2.39) when seen by using the random-effect model (I^2=^ 79.5, *P* = 00) (Fig. 8).

On the other hand, time to reach service delivering health facilities, age, residence, educational status, employment status, and discordancy were those factors which were not significantly associated with adherence to option B plus by meta-regression.

## Discussion

This review and meta-analysis was intended to find out the pooled magnitude and factors affecting adherence to option B plus PMTCT program among women in Eastern African countries. Since option B plus started in Malawi in 2011, it has brought a magnificent improvement in decreasing the transmission of HIV from mother to child. However, adherence to a lifelong ART medication becomes a challenging situation for many of the women. Various studies have been carried out on the magnitude of adherence to option B plus in Eastern African countries. But there is a lack of pooled magnitude of adherence to option B plus and factors affecting it in Eastern African countries where the consequence of HIV is big. Thus, this review was aimed to explore the overall prevalence of adherence to option B plus PMTCT program and factors affecting the adherence among women in East African countries.

In the current systematic review and meta-analysis, the overall pooled prevalence of adherence to option B plus program in the Eastern African countries was 71.88%. This finding is almost similar to three studies conducted in South Africa (68, 69, 75%) but higher than a finding from northern KwaZulu-Natal, South Africa (61%) [32–36]. It is generally recommended that adherence rates approaching 100% are needed for optimal viral suppression [37]. However, in the current meta-analysis the pooled level of adherence to option B plus was lower than the WHO recommendation which denotes; much effort is needed to improve the compliance to these medications to lower the risk of HIV transmission to the newborn and to enhance viral suppression among these mothers.

In this meta-analysis, disclosure of the HIV serostatus to the partner was positively associated with good adherence to option B plus program. Accordingly, women who disclosed their HIV/AIDS status were 4.38 (95%CI: 1.79, 10.70) times more likely to have good adherence to ART than those who had didn’t disclose their HIV/AIDS status to their partner. A study on longitudinal adherence to maternal ART in South Africa similarly revealed that non-disclosure of HIV serostatus was negatively associated with good adherence [38]. According to a study conducted in KwaZulu-Natal, and Eastern Cape, South Africa, the issue related to disclosure was a reason for sub-optimal adherence to ART medications among women [32, 33]. This implies that disclosing one’s HIV serostatus to a partner helps build trust among couples which further encourages the women to take ART medications consistently.

Partner support was also another positive factor for good adherence to option B plus program among women. In the current meta-analysis, those women who had support from their partner or husband were more likely to adhere to option B plus program than their counterparts. This finding is almost similar to a study conducted in KwaZulu-Natal, South Africa in which mothers who didn’t get support or in a quarrel with their husbands were less adherent to antiretroviral medications [33]. This proves that the role of a partner or significant other in helping women for continued compliance with ART medication is enormous. However, partner support is better insured if there is a disclosure of serostatus among couples.

Women who received counseling on the disease condition and drug adherence of ART medications were more likely to adhere to medications in the PMTCT option B plus program. A pilot study conducted on challenges of rapid ART initiation revealed that counseling and support was an important aspect for rapid treatment initiation [35]. This would imply that an integrated and continued counseling service in the provision of option B program enhances compliance with ART medications and treatment retention. There are a number of HIV-positive women who do not attend antenatal clinics which could not be benefited by counseling. Therefore, efforts have to be made to enhance the utilization of ANC at the same time.

The WHO stage of HIV/AIDS when a woman started ART medication was found to be a significant factor affecting adherence to option B plus in the current review and meta-analysis. Those women who started the ART medication while the WHO clinical stage I/II were 2.6 times more likely to have a good adherence than those stage III/IVs. Patients with advanced HIV disease could have an increased pill burden during treatment of opportunistic infections, significant fatigues in attending clinic appointments, and opportunistic infections that may create a further challenge to remembering recommendations of medication adherence [39]. This depicts the need for encouragement of women to get tested and know their HIV serostatus early before the disease advances.

### Strength and limitations of the study

The strength of the study was using various databases to search both published and unpublished studies. Moreover, this study used the PRISMA checklist and JBI-MASTARI for data extraction and appraisal which increases the quality of the study. However, since only studies published in the English language were included in the study, studies reported in other languages might be missed. Lack of estimated point prevalence of adherence level at different stages of pregnancy among included studies could be another limitation of this study.

## Conclusion

The level of adherence to option B plus PMTCT program in East African countries is generally sub-optimal. Partner support, access to counseling services, disclosure of HIV serostatus to partner or family, and clinical stage of HIV/AIDS when started ART medications were found to be significant factors affecting compliance to ART medications in the option B plus PMTCT program. Therefore, a coordinated effort is needed to raise the number of mothers to be tested, and start treatment early before the disease advances. Continuous provision of counseling services for couples on benefits of early initiation of treatment, retention in care, and adherence to medications should also be given attention. Furthermore, persuading mothers to disclose their HIV serostatus to their partner is vital which would help them in getting support and trust from their partners or family members which further improves adherence to option B plus.

## Data Availability

The data used for this study are presented within the manuscript.

## Abbreviations

AIDS: Acquired immunodeficiency syndrome
AOR: Adjusted Odds ratio
ART: Antiretroviral Therapy
CI: Confidence Interval
HIV: Human immunodeficiency Virus
JBI: Joanna Briggs Institute
PMTCT: Prevention of Mother to Child Transmission
WHO: World Health Organization
